# Feasibility and Effects of Exercise During Working Hours in Acute Hospital Staff – A Non-Randomized Controlled Trial

**DOI:** 10.1007/s10926-025-10275-6

**Published:** 2025-02-18

**Authors:** S. G. Nielsen, M. Pedersen, J. U. Toftager-Oster, C. A. Saervoll, T. K. Fischer, B. Lindegaard, S. Molsted

**Affiliations:** 1 Department of Clinical Research, Copenhagen University Hospital- North Zealand, Hillerød, Denmark; 2 Emergency Medical Services, Ballerup, Denmark; 3 Department of Public Health, University of Copenhagen, Copenhagen, Denmark; 4 Department of Clinical Medicine, University of Copenhagen, Copenhagen, Denmark; 5 Department of Pulmonary- and Infectious Disease, Copenhagen University Hospital -North Zealand, Hillerød, Denmark; 6 Centre for Physical Activity Research, Copenhagen University Hospital - Rigshospitalet, Copenhagen, Denmark

**Keywords:** Feasibility Studies, Workplace, Hospital personnel, Exercise, Resistance training, Health promotion

## Abstract

**Purpose:**

Workplace health interventions with exercise have positive effects on musculoskeletal pain and well-being at work, however, effectiveness is questioned due to low adherence. In hospitals participation is challenged by shiftwork and unpredictable workload. Our aim was to investigate the feasibility of exercise during working hours in an acute hospital, herein to estimate the health impact to guide management decisions on implementation.

**Methods:**

A clinical trial in a public hospital, offering staff supervised group-based individualized exercise with combined aerobic and strength training during working hours twice weekly for 20 weeks. Delivery, acceptance, and adherence were investigated. Subjective outcomes were social capital, well-being, quality of life, and musculoskeletal pain were assessed. Objective outcomes were blood pressure, body composition, and cardiorespiratory fitness.

**Results:**

Twenty-three percent of the employees (*n* = 617) accepted participation (92% female, median age was 50 years, 38% nurses). Adherence was 29% with no difference between employees with clinical versus non-clinical functions*.* Non-clinicians participated during working hours, while clinicians participated outside of working hours in 50% (IQR 5–87) of the sessions. Positive changes were seen in systolic and diastolic blood pressure (decreased 2.0 [0.9; 2.2] and 0.9 [0.1; 1.7] mmHG, respectively), aerobic capacity 2.3 ml/O2/min/kg [1.7; 2.9], and in waist-hip ratio, social capital, well-being, quality of life, and musculoskeletal pain.

**Conclusion:**

Exercise during working hours in an acute hospital staff was feasible, but strategies to increase acceptance and adherence are necessary for a successful implementation. Despite low adherence, the intervention was associated with improvements of physical and mental health. Registration: The study protocol has been uploaded on www.clinicaltrials.gov (NCT04988724).

**Supplementary Information:**

The online version contains supplementary material available at 10.1007/s10926-025-10275-6.

## Background

The global health workforce is challenged by high levels of sick leave, low recruitment, and retention [1]. The health of health care workers is crucial as it is associated with the efficiency of patient care as reported by the British National Health Service [2]. Thus, the World Health Organization recommends initiatives for achieving of the highest attainable standard of health and well-being in the workforce [3]. A high prevalence of musculoskeletal pain has been reported in European health care workers, both in nurses and nurse assistants [4], and in other health care professions [5]. Musculoskeletal pain is associated with psychosocial factors at work, i.e., inadequate staffing, job demands, support, time pressure, and stress [6]. Workplace health interventions with exercise training have shown positive effects on musculoskeletal pain and cardiovascular health in different populations [7] and in health care workers [8, 9]. Further, exercise has positive effects on mental health and well-being [10, 11]. The concept of Intelligent Physical Exercise Training offering supervised group-based individually tailored physical exercise training, has shown moderate adherence rates in a wide range of work areas [12] and in hospital staff [13], with the potential to improve productivity and reduce sick leave [14]. Nevertheless, the effectiveness of workplace exercise interventions is questioned due to low adherence [15]. Besides individual motivation, it has been argued that too many failures exist in workplace physical activity interventions due to insufficient design, prioritization, and implementational strategies [16, 17]. In acute hospitals, shiftwork, and low job-control may challenge implementation [6, 10], and little knowledge exists on the feasibility of exercise during working hours. A pilot trial [18] performed prior to the present study showed low to moderate adherence among hospital staff, thus the aim of the present study was to investigate the feasibility of exercise training during working hours in the entire hospital staff and to investigate the potential effects on physiological and mental health variables for future implementation.

## Methods

### Design

A feasibility trial of a non-randomized clinical intervention was conducted from April 2022 to February 2024, at an acute hospital in Denmark with approximately 3900 employees. The project initiative was funded by the hospital board with the aim of being *The Physically Active Hospital* encouraging patients, employees, and the local community to adopt physical activity. The intervention was tested in a pilot trial in one department (*n* = 214 employees) in 2021 [18], and subsequently the duration of the intervention was adjusted from 12-weeks to 20-weeks involving additionally 12 departments (employees, *n* = 2701) in 2022 and 2023. Reporting follows the CONSORT 2010 guideline for feasibility trials [19]. See Table with checklist, SI1.

### Recruitment

The hospital departments were invited in a consecutive manner to participate, and each department decided whether to join the intervention or not. Participation was voluntary and employees signed up individually on a local website at any point of time after their department had joined the project. The managements in the departments were encouraged to allow participation during working hours twice weekly, however the conditions for employees’ participation during or outside of working hours were set locally with respect to context, production, and organization of work. During the study period, the hospital administration suggested that the departments should offer compensation if participation during working hours was not possible, i.e., payment hour to hour or planned leave. Information about the intervention was provided at staff meetings, in newsletters, and posters.

### Ethics

Written and oral information contained the purpose of the intervention, and that participation was encouraged twice weekly for 20 weeks after agreement with local managers. Assurance was given that all data would be anonymized and available for researchers only, thus managers would have no access to participants’ personal data. Data collection was approved by the Data Protection Agency (P-2022–198). Written consent was collected. The study was conducted in accordance with the Helsinki declaration and the rules of The Danish National Committee on Health Research Ethics Journal (H-21038302). The study was registered and the protocol uploaded on www.clinicaltrials.gov (NCT04988724). The duration of the trial was extended two months to allow for follow-up assessments of participants entering the trial in the autumn of 2023.

### Intervention

Supervised exercise in a modified version of the concept of Intelligent Physical Exercise Training was provided, to improve fitness, muscle strength, reduce musculoskeletal pain as well as to improve adherence to physical activity and thus reduce sickness absence, as described by Sjøgaard et al. [12]. In short, sessions were supervised by exercise coaches and consisted of a 5-min warm up, 12-min of aerobic high intensity interval training, and 12-min of individualized resistance training. Thirty exercise sessions were scheduled, and available at different hours between 7.00 AM and 16.15 PM Mondays through Fridays, accommodating employees with different working hours and preferences. Participants were registered as members on the website SportMember.com (SportMember.com, Aarhus, Denmark), and using the mobile application, exercise sessions were easily available for sign up. At each session the participants registered if they participated: a) within working hours, b) outside of working hours, or c) outside of working hours with time compensated. The exercise sessions were provided on an outdoor fitness area (Kompan©, Odense, Denmark) on the hospital area accessible within a five-minute walk from all departments. If the weather did not allow outdoor activity an indoor gym was accessible. Individual consultations were provided before and after the 20-week exercise intervention, in which participants completed questionnaires and health measurements. Personal goals were established, and an individualized resistance training program constructed. After completing the intervention, employees were encouraged to continue exercise participation on the terms decided locally in their respective departments.

### Data Collection

All data were stored in REDCap (REDCap 10.6.18, Vanderbilt University, USA).

Descriptive data were collected at baseline and included age, sex, profession, job function, department as well as self-reported physical capacity rated on a five-item Likert scale from ‘really poor’ to ‘excellent’ [20]. Delivery was evaluated as the number of exercise sessions and occupancy rate. The acceptance by departments and employees was evaluated as the number of departments participating, as well as the relative distribution of employees and professions.

#### Self-Reported Outcomes

Social capital was assessed within team and in relation to other teams/departments using the Danish Social Capital Questionnaires [21] with questions about collaboration, communication, recognition, support, and atmosphere. Well-being at work was assessed with the Danish version of the Utrecht Work Engagement Scale [22]. Questions concerned energy, enthusiasm, concentration, and motivation. Answers provided on Likert scales were used to calculate mean scores between 0 and 100. Health-related quality of life was assessed using the EQ-5D-5L [23], assessing mobility, self-care, usual activities, pain/discomfort, and anxiety/depression. Values from 1 to 5 were converted to a total score according to standard procedures. Musculoskeletal pain was assessed with a numeric rating scale from 0 to 10 on five body areas: neck/shoulder, back/upper back, low back, arms/hands, and legs/feet. Self-reported physical activity was assessed with The International Physical Activity Questionnaire (IPAQ) [24]. Minutes spent with low, moderate, and high intensity physical activity were used to calculate metabolic equivalents per week. Unintended events and the consequences were registered.

#### Objectively Measured Outcomes

Blood pressure was measured three times using a cuff (Microlife AG, Switzerland) on the left arm after sitting quietly for 15 min. The systolic pressure (mmHg) from the lowest measurement was used with the associated diastolic measurement. Height was measured in a stadiometer (Charder Height Measurement HM200P, Charder Electric, Taiwan), and body weight was measured clothed without shoes using an electronic weight (Seca 799, Seca GmhB, Germany). Body Mass Index was calculated (kg/m^2^). Waist and hip circumferences (cm) were measured using a tape measure in the standing position. The waist was measured midway between the lowest rib to the anterior superior iliac spine after exhaling with arms by the side, while the hip was measured at the widest part. The waist-to-hip ratio was calculated using waist divided by hip circumference. Aerobic capacity was estimated using the 1-point Aastrand test [25]. The test was performed on a bicycle ergometer (Monark E828, Vansbro, Sweden), and heart rate was tracked with a heart rate monitor (Polar H1 Pro, Kempele, Finland). Muscle strength testing was planned, however, due to technical issues with the equipment, the test was omitted.

### Statistical Analyses

Adherence was evaluated as the number of exercise sessions per participant, and this was analyzed in relation to clinical or non-clinical functions, age-groups, and self-reported physical capacity. Changes in health outcomes were assessed for participants with at least one exercise session during the 20-week intervention period. Variables were inspected for normal distribution using Q-Q plots. Student’s paired *t* test was used to investigate changes of normally distributed continuous data, and Wilcoxon signed rank test when data were not normally distributed. One-Way Anova was used to test differences between multiple groups when data were normally distributed, and the Kruskal–Wallis test when they were not. Post hoc analyses were performed on overall significant differences. Data are presented as number and percentages, median (interquartile range (IQR)), mean ± standard deviation (SD), or mean [95% confidence interval (95% CI)]. The analyses were performed using the IBM SPSS 25 program (IBM Corp, Armonk, NY). P-values with a significance level of *p* < 0.05 are given to inform on the responsiveness of the measurement tools for future implementation.

## Results

### Participants

The participants were predominantly women with a median age of 50 years (IQR 39–57), and nurses were the most common staff constituting 38% of all, followed by clerks and doctors (11%) (Table 1).

**Table 1:** Baseline characteristics of participants with ≥ 1 exercise session

Variable	Participants (*n* = 465)
Age (years)	50 (39–57)
Sex (women)	426 (92%)
Body mass index (kg/m^2^)	25 (22–28)
Blood pressure (mmHg) (n = 458)	
Systolic	118 ± 17
Diastolic	77 ± 10
Professions (n)	
Nurses	178 (38%)
Clerks and academics	57 (12%)
Secretaries	49 (11%)
Doctors	49 (11%)
Medical laboratory technologists	24 (5%)
Physiotherapists	18 (4%)
Midwifes	16 (3%)
Nursing assistants	14 (3%)
Occupational therapists	11 (2%)
Service assistants	4 (1%)
Others	45 (10%)
Job function (n)	
Clinical	288 (62%)
Non-clinical	177 (38%)

Data are presented as median (IQR), number (%), or mean ± SD. *n* = numbers

### Delivery

The participants participated in 20 (20–20) weeks of training. An average of 119 exercise sessions were delivered per month with a mean occupancy rate of 3.8 participants per session, totaling 10,056 exercise sessions completed by 465 employees, which included participants who continued to exercise after their 20th week follow-up test.

### Acceptability

**Fig. 1:**
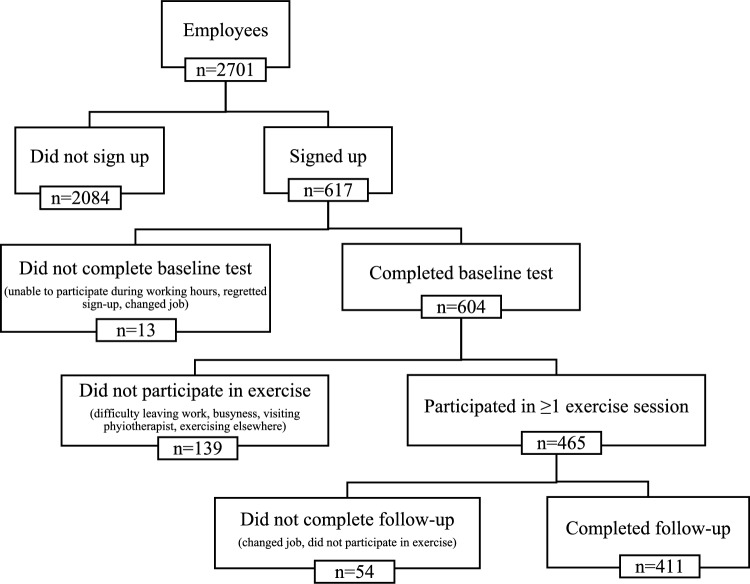
The flow of sign up, participation and completion of testing and exercise participation. In parentheses are reasons given. Abbreviations: *n* = numbers

Twenty-three percent of employees from 12 departments signed-up and 88% completed their posttest after at least one training session (Fig. 1). Eight of the participating departments were clinical, two were administrative, one was technical, and the Red Cross participated with volunteers. Five departments joined the intervention in March 2022, four in January 2023 and three in August 2023. The departments with highest relative acceptance (sign-up in percent of department population) were Department of Clinical Research (79%) and The Administration (62%), while The Red Cross (7%) and Facility Management (3%) had the lowest acceptance rates.

### Adherence

Participants with at least one training session participated on average 12 (5–22) times during the intervention period, however for participants completing their 20-week follow-up session the participation rate was 15 (7–24) times. Participants aged 30–39 years had the lowest adherence as compared to those < 30 years (*p* = 0.025), 50–59 years (*p* = 0.003), and above 60 years (*p* < 0.001). Participants who rated their physical capacity “Very poor” at baseline had the lowest adherence rate, 14% lower than participants who rated it “Average” (*p* = 0.009) and 19% lower than those rating it “Good” (*p* < 0.001), respectively. There were no differences in adherence when the employees were stratified into clinical and non-clinical functions, professions, or sex (Table 2).

**Table 2:** Baseline characteristics in relation to training adherence

Variables	*n*	Completed sessions	*p*
Exercise sessions per participant (%)	465	28.9 (12.5–55.0)	
Clinical or non-clinical function (%)	465		0.858
Clinical	288	30.0 (12.5–52.5)	
Non-clinical	177	26.0 (12.5–57.5)	
Age groups (%)	465		0.005
22–29 years	31	39.5 (16.7–62.5)^a^	
30–39 years	94	20.7 (8.1–42.7)	
40–49 years	95	22.5 (12.5–55.0)	
50–59 years	171	31.3 (15.0–60.0)^b^	
60–76 years	74	37.2 (20.8–57.5)^c^	
Self-reported physical capacity (%)	463		0.001
Excellent	3	10.0 (2.5-)	
Good	84	37.8 (18.8–70)^d^	
Average	231	(15.0–58.3)^e^	
Poor	117	(10.7–49.0)	
Really poor	28	18.8 (12.5–25.0)	

Data are presented as median (IQR). Relative number (%) of completed exercise sessions during the 20-week intervention. *n* = number, ref = reference. ^a^ vs. 30–39 years *p* = 0.025; ^b^ vs. 30–39 years *p* = 0.003; ^c^ vs. 30–39 years *p* < 0.001; ^d^ vs. Poor *p* < 0.001; ^e^ vs. Poor *p* = 0.009

Of the 6072 sessions completed within the 20-week intervention period, 60% took place during working hours, and 37% of the sessions were outside of working hours with or without compensation (3% missing data). Clinicians participated before or after work in 50% (5–87) of the sessions, whereas non-clinicians participated during working hours, 100% (83–100).

### Self-reported outcomes

Positive changes were seen in social capital between teams/departments, in well-being at work and in health-related quality of life, in which the improvement was in the pain/discomfort dimension (*p* = 0.002) (Table 3).

**Table 3:** Self-reported and objectively measured outcomes at baseline and follow-up

Variables	n	Baseline	Follow-up	*p*
Social Capital (0–100)				
Within team	411	72.2 [71.0; 73.3]	72.9 [71.6; 74.1]	0.128
Between teams/departments	410	63.5 [62.3; 64.7]	65.3 [63.9; 66.7]	0.006
Well-being at work	411	68.2 [67.1; 69.4]	71.5 [70.4; 72.6]	< 0.001
Health-related quality of life	411	0.952 (0.906–1.000)	0.952 (0.911–1.000)	< 0.001
Mobility	411	1 (1–1)	1 (1–1)	0.148
Self-Care	407	1 (1–1)	1 (1–1)	0.796
Usual activities	404	1 (1–1)	1 (1–1)	0.885
Pain/discomfort	408	2 (1–2)	2 (1–2)	0.002
Anxiety/depression	408	1 (1–1)	1 (1–1)	0.201
Physical activity level(MET-min/week)	407	2514 (1470–3756)	2580 (1476–4110)	0.059
Blood pressure (mmHg)	364			
Systolic		118.4 [116.8; 120.0]	116.4 [114.8; 118.0]	< 0.001
Diastolic		77.5 [76.5; 78.5]	76.6 [75.5; 77.6]	0.030
Body mass index (kg/m^2^)	384	24.7 (24.4–28.3)	24.8 (22.4–28.1)	0.033
Waist (cm)	382	85.5 (78.0–96.0)	84.8 (77.0–93.0)	< 0.001
Hip (cm)	382	104.0 (99.0–111.0)	104.0 (98.0–110.0)	< 0.001
Waist:Hip ratio	382	0.83 [0.82; 0.83]	0.82 [0.82; 0.83]	0.002
VO_2Max_ (ml/O_2_/min/kg)	349	34.9 [33.9; 36.0]	37.2 [36.1; 38.3]	< 0.001

Data are represented as median (IQR) and mean [95% CI]: *MET* = Metabolic equivalent, *VO*_*2Max*_ = Maximal aerobic capacity

**Fig. 2:**
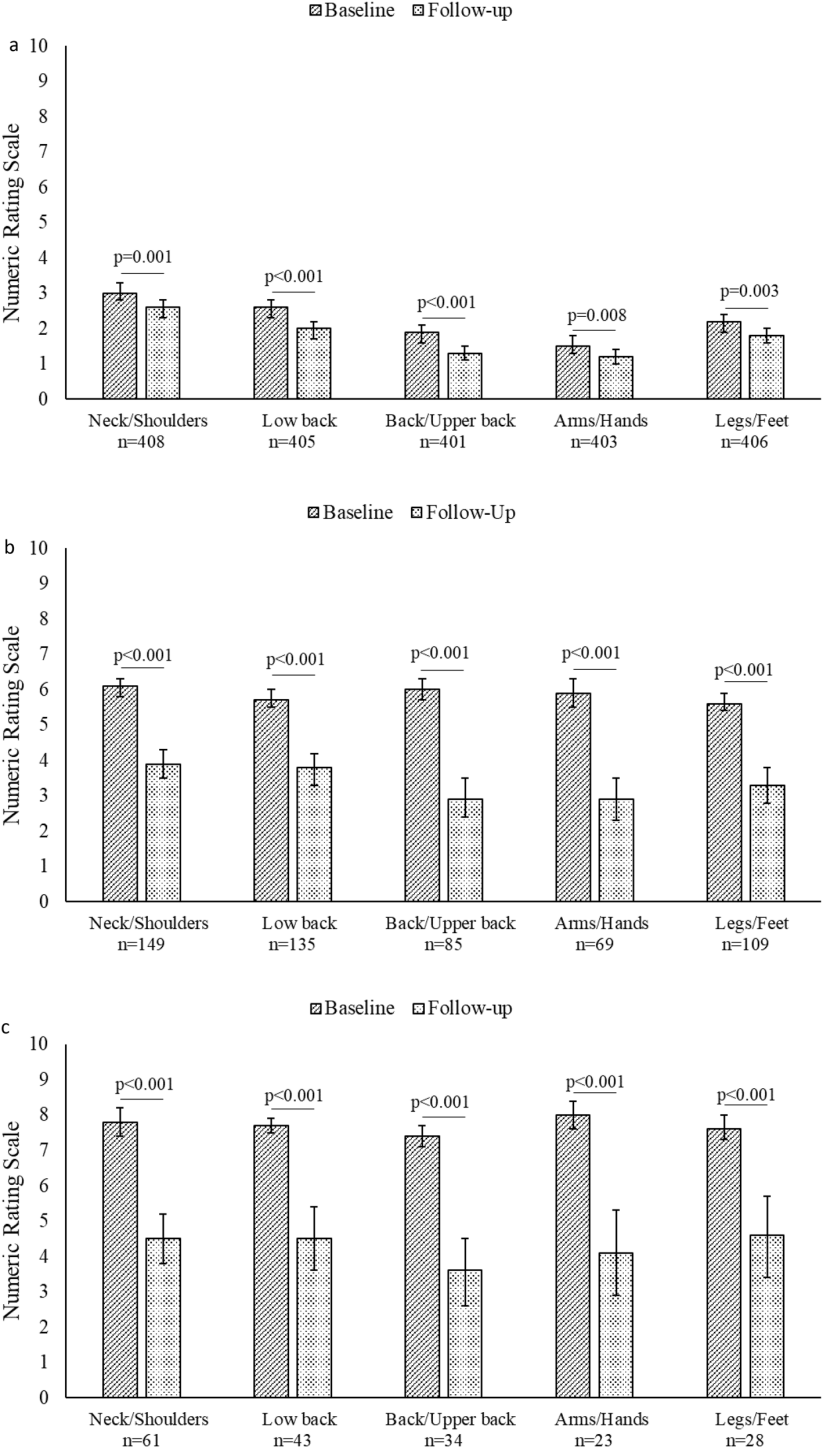
Changes in the pain dimension for participants with ≥ 1 exercise session. a, baseline and follow-up scores for pain dimensions. b, Baseline and follow-up scores for participants with baseline pain > 3. Fig. c, Baseline and follow-up scores for participants with baseline pain > 6. Error bars represent 95% CI

Positive changes were seen in the pain dimension in all body areas and the improvements were also significant in the subgroups of participants with moderate pain at baseline (numeric rating scale > 3) and severe pain (> 6) at baseline (Fig. 2). Self-reported physical activity level remained unchanged.

### Objectively Measured Outcomes

Systolic and diastolic blood pressure were reduced 2.0 [0.9; 2.2] and 0.9 [0.1; 1.7] mmHg, respectively. Body mass index increased 0.1 kg/m^2^ (− 0.5–0.3), while waist and hip circumferences, and the waist-hip ratio decreased. The aerobic capacity increased 2.3 ml/O_2_/min/kg [1.7; 2.9] (Table 3).

### Adverse Events

Twenty adverse events occurred, causing three participants to terminate their participation: two females experienced knee pain during a low impact exercise class, and one female had back pain after strength training. The remaining incidents were temporary soreness in knees, backs, and elbows not affecting participation.

## Discussion

The most important finding was that the exercise intervention was accepted by a moderate number of employees, who participated with a rather low adherence. Nevertheless, the intervention was associated with important positive changes in blood pressure, waist-hip ratio, aerobic capacity, social capital, well-being, health-related quality of life, and musculoskeletal pain.

The moderate acceptance and adherence were similar to previous findings in the pilot study prior to this feasibility study [18], and the reasons may be several. First, the employees may have lacked support from their managers, limiting their motivation to sign up and their ability to participate as described during the pilot study [26]. Qualitative investigations in the present study revealed that some department managers hesitated their engagement and support of the intervention due to organizational and operational barriers in clinical practice [27]. Systematic reviews have addressed the essential role of managers showing leadership in implementation of workplace interventions [9, 28]. We found that clinical staff more often participated outside working hours, in opposition to non-clinical personnel participating during working hours. While many were offered a compensation for their participation outside working hours, it contradicts with the general idea that workplace exercise during working hours could eliminate barriers, such as time or family obligations.

With regards to work-life-balance, we showed that participants aged 30–39 years had the lowest rate of participation. We speculate, that parents with young children and professionals in their early carriers, may have difficulty in the prioritization of personal matters like exercise. If this is the case, it confirms the relevance of workplace interventions during working hours aiming to improve physical activity in this decade of life. Our experiences are that the maturation of clinical practice in which exercise participation is accepted and prioritized requires time and effort. For implementation of exercise in acute hospitals, strategic work with the development of health literacy responsiveness is warranted [29].

A second reason for low participation and adherence may be, that individuals with impaired physical function hesitated or withdrew from participation. We found that participants reporting a ‘very poor physical capacity’ at baseline, had a lower attendance rate compared to participants with ‘average’ or ‘good’ physical capacity. Due to the non-randomized design, we cannot exclude the risk of volunteer bias, however, more than half of the participants reported having musculoskeletal pain at baseline, indicating a motivation to take up exercise despite pain. This underscores the importance of offering supervision by exercise professionals, who can motivate these participants and tailor the exercises, so that adverse events are prevented. The concept of Intelligent Physical Exercise Training with individualized exercise programs was provided and proved its worth with very few adverse events registered. To improve acceptance, and reduce inequity among professions, strategies must be made to handle organizational, interventional, and individual barriers toward exercise participation [30].

A third logistic reason for low adherence, may be that over a 20-week intervention period most employees have taken one or more weeks of vacation.

The objective and subjective outcome measures used in the study detected positive changes related to exercise training as could be expected. The change in the systolic blood pressure of two mmHg is clinically relevant for reducing the risk of cardiovascular events in middle aged healthy adults [31]. The cycling tests showed increases in the VO_2max,_ anticipated to have substantial health benefits [32]. No data on diet were collected, and positive dietary changes could have influenced the outcomes. Whether these changes are effects of the present intervention cannot be proven in the non-randomized design, however, if general lifestyle changes occur with such a health promotion initiative in the organization, the potential benefits should not be neglected.

A very important finding was the reductions in pain in all body parts, and these changes were more pronounced the greater the pain was at baseline. The high prevalence of musculoskeletal pain is in accordance with reports on health care workers and associated with sick leave [6, 33]. Thus, pain reductions are essential on an individual level for the general health and quality of life, and on a societal level for reducing expenses with low productivity in hospital staff with a physically demanding work [33].

Finally, very important findings were the positive changes in social capital, well-being at work, and quality of life emphasizing a potential benefit on mental health from group-based physical activity in an informal social atmosphere among staff. No cost–benefit analysis has been made, however for comparison, calculations on direct and indirect costs were performed on a smaller scale intervention in the pilot study [34].

### Limitations

The lack of a control group is a limitation of evidence and does not allow conclusions on effects. Volunteer bias may have occurred in the recruitment, resulting in a skewed representation of participants. Lack of blinding may have influenced test results, although no recall-bias is anticipated. The repeated blood pressure measurements may have been influenced by variations in time of day, diet and physical activity. The strengths of the study were the use of validated tests and a great number of participants.

## Conclusion

Supervised exercise offered to employees at an acute hospital was feasible, however strategies targeting a higher participation as well as adherence rate are essential prior to further implementation of the program. Even with a low adherence, a promising potential exists to improve the physical and mental health.

## Supplementary Information

Below is the link to the electronic supplementary material.

Supplementary file1 (PDF 4122 KB)

## Data Availability

Data availability: The datasets generated and analyzed during the current study are available from the corresponding author on request.
